# Characterization of Domestic Violence among Patients Consulting a French Psychiatric Emergency Department

**DOI:** 10.3390/healthcare11060832

**Published:** 2023-03-12

**Authors:** Laetitia Leveque, Stavroula Papadodima, Ryan Toutin, Mathieu Fraigneau, Eric Baccino, Laurent Martrille

**Affiliations:** 1 EDPFM, Department of Forensic Medicine, University of Montpellier, CHU Montpellier, 34000 Montpellier, France; l-leveque@chu-montpellier.fr (L.L.);; 2 Department of Forensic Medicine and Toxicology, School of Medicine, National and Kapodistrian University of Athens, M. Asias 75, 11527 Athens, Greece; 3 Service de Médecine Légale, Centre Hospitalier Universitaire Rangueil, 31059 Toulouse, France; 4 Department of Emergency Psychiatry and Acute Care, CHU Montpellier, 34000 Montpellier, France

**Keywords:** domestic violence, psychiatric disorders, emergency, forensic clinical examination, abuse

## Abstract

The aim of the present study was to identify the prevalence of domestic violence among patients attending a French psychiatric emergency department and its association with psychiatric disorders. This retrospective study was performed, including all patients examined in the psychiatric emergency department of the Lapeyronie University Hospital of Montpellier (France) in the daytime from 1 July 2021 to 31 October 2021. A total of 152 patients were eligible during this study period. The prevalence of domestic violence was 38.2% (*n* = 58) overall. The percentage of female victims of domestic violence was higher than that of male victims (47.6% vs. 17.0%, *p* < 0.001). Among the 58 victims of domestic violence, 20.7% reported psychological abuse, 17.2% physical abuse, 3.4% sexual abuse, and 58.6% multiple forms of abuse. The risk of suicide attempt and anxiety disorder among the female patients was associated with domestic violence (*p* = 0.006, OR = 7.24, and *p* = 0.010, OR = 0.16). Our study showed that the psychiatric population should be identified as a population at risk for domestic violence, especially when the patient is female and suffers from anxiety disorders or if she has performed a previous suicide attempt.

## 1. Introduction

Domestic violence or intimate partner violence is a significant health and social problem that the WHO has considered a public health problem, both among women [1] and men [2]. Domestic violence is defined as abusive or violent behavior between people who are or have been intimate partners or family members. Abuse can be psychological, physical, sexual, financial, or emotional. This encompasses all forms of abuse, including financial, emotional, physical, and sexual [3]. Abuse in the context of domestic violence can occur among people with any gender identity and sexual orientation and does not require sexual intimacy [4]. 

In France, the term “domestic violence” refers to violence within a couple, as well as violence against the couple’s children. Domestic violence is also considered in cases of former spouses, partners, or civil union partners. According to Art. 515-9, Law n° 2010-769 of 2010, “When violence within the couple or by a former spouse, a former partner under a civil solidarity pact or a former partner, endangers the person who is the victim, and/or one or more children, the Family Court may issue an emergency protection order”. Forced sex with a partner is not justified by the couple’s relationship. Moreover, violence within a couple in the French legal system is an aggravated criminal offense with increased penalties [5].

When the perpetrator is a current or former partner, the domestic violence is characterized as Intimate Partner Violence [6,7]. The concepts of domestic violence and intimate partner violence are very similar to one another, and both presuppose experiencing violence from very close individuals, and they are frequently used interchangeably in the literature; however, domestic violence includes cases of violence from other family members, as well as cases of mutual violence in the family [8].

Especially violence against women is a serious issue for both public health and human rights, as it is a pervasive, unreported epidemic in all human cultures [9,10,11]. Estimates published by the WHO indicate that globally, about one in three (30%) women worldwide have been subjected to sexual and/or physical abuse by their intimate partner or sexual abuse by another perpetrator in their lifetime [6,12]. This prevalence varies among studies in men as well; percentages ranging from 3.4% to 20.3% have been reported [2,13]. In France, the prevalence of lifetime physical and/or sexual intimate partner violence against women has been reported to be 26%; physical and/or sexual intimate partner violence in the last 12 months was 5%, and lifetime non-partner sexual violence was 9% [14].

Domestic violence has been reported as a threat to women’s physical and mental health. Chronic pain [15], injuries [15], pregnancy complications (such as reduced birth weight, premature birth, infection, and miscarriage/abortion) [16], sexually transmitted diseases, such as HIV [17], menstrual disorders [18,19], and cardiovascular problems [20], have been reported as the most frequently reported physical health outcomes of domestic violence. Mental health problems have also been cited by several authors and include post-traumatic stress disorder [21,22], depression [23,24], functional symptoms [25], exacerbation of psychotic symptoms [26], anxiety [22,23,24], low self-esteem, psychosomatic complaints [27], substance misuse [28], self-harm, suicidal ideation, and suicide attempts [12,29,30,31,32]. Long-term exposure to domestic violence may also have a significant impact on the duration of mental disorders. Golding [33], in his systematic review, showed that the rates of depression declined over time after the cessation of abuse and that the severity and prevalence of depression are related to the severity and duration of abuse. 

However, the link between domestic violence and psychiatric disorders is bidirectional—domestic violence can damage mental health, but psychiatric disorders can also render an individual more vulnerable to domestic violence [34]. Indeed, the prevalence of domestic violence is frequent in the psychiatric population, with percentages ranging from 30% to 60% [34]. Oram et al., in their systematic review, found a median prevalence of lifetime partner violence of 30% among psychiatric female in-patients, 33% among female out-patients, and 32% among male patients. These numbers, which are higher than in the general population, may be explained by certain particularities of this population, such as an increased vulnerability to domestic violence and an association with both risk and chronicity of mental disorder [35]. 

The healthcare service appears uniquely positioned to prevent and intervene in domestic violence [36]. Direct questioning may be a useful and effective tool for eliciting disclosures from victims of domestic violence [34,37]. Psychiatric emergency departments are specially offered for this purpose [38].

The aim of the present study was to identify the prevalence of domestic violence among patients of both sexes attending a French psychiatric emergency department. Additional aims were to determine the frequency of each type of domestic violence, the timing of violence, the associated psychiatric disorders, and the proportion of patients who file a complaint. 

## 2. Materials and Methods

This study was carried out in the psychiatric emergency department of the Lapeyronie University Hospital in Montpellier (France). The emergency department is open all week, 24 h a day. During the day (8:30 a.m. to 7:00 p.m.) and from Monday to Friday, the emergency department is supported by a team of experienced psychiatrists, as well as psychologists, and social workers, who intervene when necessary. During the examination of patients, the psychiatrist asks questions about domestic violence, and psychologists and social workers familiar with domestic violence are available to assist the physician when confronted with it. If domestic violence is identified, the psychiatrist will, where possible, clarify the time and form of the violence and may also ask whether the patient wishes to make a complaint or has already made one. All answers are collected in the patient’s electronic file (Dx urgency software). These practices are in line with the public health recommendations in France from 2019 regarding the need for better detection and management of domestic violence [39]. At night and on the weekends, different psychiatrists from different departments may attend. There are no psychologists or social workers during the night, and the search for domestic violence is not performed in a homogeneous and systematic way. For these reasons, we collected only the computerized files of patients referred during the day. In the psychiatric emergency department of the Lapeyronie University Hospital of Montpellier, patients can be referred directly to a psychiatrist, psychologist, or general practitioner at their request. When examined by a psychiatrist, they are not obliged to undergo a physical examination in the emergency department unless the psychiatrist requests it after his/her own examination. Sometimes, when a patient visits the emergency department for a physical examination, the emergency physician may seek the advice of the psychiatrist, who will add his/her comments to the patient’s record.

Thanks to this organization, we conducted a retrospective, observational, single-center assessment of domestic violence among patients consulting the psychiatric emergency department. All patients examined in the psychiatric emergency department of the Lapeyronie University Hospital of Montpellier (France) from 8:30 a.m. to 7:00 p.m. from 1 July 2021 to 31 October 2021 were included in this study. Minors (age < 18 years) and patients with altered mental status due to any cause at the time of examination (under the influence of alcohol, drugs, sedatives, or acute psychiatric disorders), as well as patients previously examined, were excluded. 

The data collection from the patients’ computer records was performed by a physician, a resident in forensic medicine. The collected data included the presence or absence of domestic violence, timing (current, in the past, chronic), form of domestic violence (physical, sexual, psychological, or multiple, i.e., two or more types), the filing of or the intention to file a complaint, and the existence of a psychiatric disease, including addictions (excluding tobacco smoking) recorded during the consultation.

Psychiatric disorders were classified into the following categories based on DSM-V criteria:

- Mood disorders: depressive disorder, bipolar type I and II;
- Anxiety disorders: generalized anxiety; panic disorder; and phobic disorder;
- Psychotic disorders: schizophrenia; persistent delusional disorder; brief psychotic episode;
- Personality disorders;
- Addictive disorders (alcohol, cannabis, ecstasy, amphetamine, methadone, opiate, synthetic drugs);
- Disorders related to trauma and stress: posttraumatic stress disorder and adjustment disorders;
- Others: obsessive–compulsive disorder; dissociative disorder.

The “no psychiatric disorders” category included the cases in which no psychiatric diagnosis was established or the mental symptoms were attributed to a somatic disorder (for example, dementia). 

The existence or previous suicide attempt was also recorded.

All statistical analyses were carried out via the SPSS version 25.0 (IBM Corp. Released 2017. IBM SPSS Statistics for Windows, Version 25.0. Armonk, NY, USA: IBM Corp). The bivariate analysis of the qualitative variables was carried out via the Fisher’s Exact Test, while quantitative variables were analyzed via the Student’s *t*-test. A logistic regression was carried out via the Enter method. The threshold of statistical significance (*p*-value) was set as 0.05.

## 3. Results

### 3.1. Prevalence, Time, and Type of Domestic Violence

Out of the 254 patient files, 152 (60%) were eligible during the study period (105 females and 47 males). Fifty-eight patients (38.2%) reported domestic violence. The percentage of female victims of domestic violence was higher than male victims (47.6% vs. 17.0%, *p* < 0.001). The mean age of males subjected to domestic violence was lower (33.88 years) than males not subjected to domestic violence (44.56 years) (*p* = 0.029). On the contrary, female victims of domestic violence were older compared to females who were not victims (42.3 vs. 36.2, *p* = 0.043). The above results are presented in Table 1.

Among the 58 patients who experienced domestic violence, 20.7% reported psychological abuse (*n* = 12 patients), 17.2% physical abuse (*n* = 10), 3.4% sexual abuse (*n* = 2), and 58.6% multiple forms of abuse (*n* = 34). Cases of multiple abuse are presented in Table 2. This domestic violence occurred in the past in 75.9% (*n* = 44 patients); it was current in 15.5% (*n* = 9), and it was chronic in 8.6% (*n* = 5). 

### 3.2. Frequencies of Associated Psychiatric Disorders

Psychiatric disorders, according to reported domestic violence, showed non-significant differences for all disorders except for anxiety disorders in the female population (Table 3). More specifically, the women who reported domestic violence had a lower frequency of anxiety disorders (*p* = 0.027). On the other side, in the above female subpopulation, the frequency of previous suicidal attempts was higher (*p* = 0.045). The logistic regression analysis found that the risk of suicide attempt was associated with domestic violence (*p* = 0.021, OR = 5.29, 95% CI 1.28–21.86) (Table 4). 

### 3.3. Frequency of Complaints

Out of the 58 patients who reported domestic violence, 20 patients wished to file or had filed a complaint (34.5%); 9 of them were patients who were experiencing current domestic violence. Out of the 35 patients who did not want to file a complaint, the main reasons were fear of consequences, separation or divorce, or believing that the violence was deserved.

### 3.4. Other Findings

Sometimes the patient was not a victim of domestic violence but spontaneously mentioned other types of violence. Out of the 152 patients, 11 (7.2%) reported that they were victims of intra-family violence. Nine (6%) reported not being victims but perpetrators of domestic violence (including three cases of mutual violence; these three cases were included in the 58 patients reporting domestic violence). Finally, two patients (1.3%) reported having been sexually assaulted by a stranger. 

## 4. Discussion

We aimed to evaluate the prevalence of domestic violence in patients who consulted the psychiatric emergency department of Montpellier (France). Our study found that 38.2% of the patients who visited the psychiatric emergency department between July and October 2021 reported domestic violence. 

Domestic violence is recognized as common among psychiatric patients. Sahraian et al. found a prevalence of 52.8% among 250 female psychiatric patients in Iran [40]. Dallay, in her practice thesis on psychiatric disorders and domestic violence (“PréOCUP investigation”, in Bordeaux, France), found a prevalence of 23.9% [41]. Howard et al., in their review that included 22 studies, found that percentages for lifetime reported domestic violence ranged between 34% and 63% for females and between 14% and 48% for male psychiatric in-patients. In a population of psychiatric out-patients, the percentages were 15–90% and 0–13% for females and males, respectively. Oram et al., in their extensive systematic review that included 42 studies, found a prevalence between 30% and 33% in women and men, in- or out-patients [35]. 

Studies on domestic violence have been conducted in emergency medical services. In the United Kingdom, Boyle et al. found a prevalence of 22.4% in women and 22.1% in men in an emergency department [31]. In France, Lejoyeux et al. found a prevalence of 18% and pointed out that patients suffering from domestic violence presented a psychiatric disorder significantly more often than controls [42]. Another study performed in the United States by Scholle et al. estimated that the prevalence of physical abuse among depressed women was 55% [43]. 

Domestic violence appears to be more frequent in the psychiatric population for many reasons. Golding explained that “the literature suggests that the prevalence of mental disorders among women who have been battered is high. This evidence is consistent with the hypothesis that intimate partner violence serves as a risk factor for mental health problems” [33]. The link between domestic violence and psychiatric disorders could be akin to a vicious circle. Even if a direct causal link cannot be proved, a correlation may exist. Psychiatric disorders could render a woman more vulnerable to domestic violence, and domestic violence could lead to psychiatric disorders. Thus, even if there is no typical profile for being a victim of domestic violence [23], having a psychiatric disorder could be a risk factor. 

In our study, the majority of the victims had sustained multiple forms of abuse (58.6%), where 20.7% had sustained strictly psychological violence, 17.2% strictly physical violence, and 3.4% strictly sexual violence. The patients reported domestic violence that had occurred in the past (75.9%), was current (15.5%), or chronic (8.6%). In the literature, there is no particular trend regarding the form of violence, although psychological violence appears to be the most prevalent [44,45]. Oram et al., in their extensive systematic review, analyzed physical, sexual, and psychological forms of domestic violence [35]. They showed that lifetime physical violence occurred in 26% of female in-patients and 18% of male in-patients, and psychological violence in 94% of female in-patients. The respective percentages for psychiatric female and male out-patients were 8–80% and 2–6% for physical violence, and 9–72% for psychological violence (females). The above review showed that studies on out-patients regarding sexual violence were limited to females and reported highly variable estimates. Studies on women visiting psychiatric emergency departments showed a prevalence of adult lifetime domestic violence ranging from 42% to 60% among female and 8% among male patients [34]. Regarding the timing of the violence, it is worth mentioning that chronic violence or repeated/continuous violence has a greater impact on the victim’s psyche, as explained by Walker [46]. 

In our study, a positive association was found between domestic violence and previous suicide attempt. Regarding previous suicide attempts, Boyle et al. stated that the presence of deliberate self-harm is associated with personality disorders (mostly those related to childhood abuse and/or neglect) and that it is also connected with an increased risk of entering an abusive relationship [31,32]. Despite this bias, it cannot be excluded that being a victim of domestic violence is a risk factor for suicide attempts. In fact, several authors have demonstrated the above link. Cavanaugh et al. suggested that one in five adult female victims of intimate partner violence in their study in the United Kingdom have threatened or attempted suicide during their lifetime [30]. Rasmussen et al. investigated the prevalence of intimate partner violence victimization in women presenting to the emergency department in a suicidal crisis, and they found that 61% of women with a recent suicide-related presentation to the emergency department reported intimate partner victimization, of whom 36% experienced recent victimization [29]. Being a victim of multiple violence has also been shown as a risk factor for attempting suicide [12]. 

The presence of anxiety disorder has been found in 76% of female victims of violence examined in a medicolegal department in Morocco [23]. In a large retrospective study in the United Kingdom, anxiety was found to be associated with exposure to intimate partner violence in the female population [24]. A study from Spain showed that women submitted to physical and/or psychological intimate partner violence presented a higher severity and incidence of anxiety and depressive symptoms, posttraumatic disorder, and suicidal ideation than control women [22]. Consequently, our finding regarding the negative association between anxiety disorders and domestic violence was rather surprising. It should, however, be mentioned that physical and mental health consequences may be linked in different ways with various types of abuse among women of different ethnical origins, as shown by Lacey et al. in their study of black, Hispanic, and white women in the USA [47]. The influence of social and contextual factors on women’s health and overall well-being should also be taken into account when trying to explain the relationship between domestic violence and the appearance of a mental disorder or symptom. Several approaches have been reported regarding the way that people deal with stressful stimuli, such as abuse. In the problem-solving-oriented coping method, the individual concentrates on the problem and its solution. In the emotion-oriented coping method, on the other hand, the individual focuses all of his/her efforts on decreasing his/her emotions [48]. A third approach has also been described, the so-called avoidance approach, which involves diverting attention by amusing and engaging in social activities or absorbing a new activity [49]. Female victims of domestic violence have been reported to apply emotion-oriented strategies to cope with domestic violence or its outcomes more than problem-solving strategies [50,51]. Adopting a coping strategy focusing on ameliorating feelings rather than solving problems may be partly responsible for a diminished level of declared anxiety among the victims of domestic violence in our sample. However, further research, including a detailed recording of several, probably confounding, parameters, is needed before drawing any conclusions on the matter.

In the present study, a statistically significant association between the other psychiatric disorders and the presence of domestic violence was not found, probably because of the small size of our sample. On the other side, as already mentioned, specific characteristics of several populations may affect the link between domestic violence and psychiatric problems.

In this study, 34.5% of the patients wanted to file or had filed a complaint. Among the above patients, nine were victims of current domestic violence, and all of them decided to file a complaint. The above proportion is quite low, in accordance with observations in the general population. Sanz-Barbero et al. found, in the context of a Spanish survey on gender violence, that 72.8% of women exposed to domestic violence had not reported their aggressor. The most frequent reasons for not reporting were underestimation of the situation and fear and lack of trust in the reporting process. The main reasons for withdrawing the complaint were the cessation of the violence and their submission to fear and threats [52]. Saavedra et al. estimated that only 22% of women who report having been victims of intimate partner violence file a formal complaint in Chile. They explained that the main reasons for not reporting episodes of intimate partner violence included believing that the violent episodes were not severe, feeling ashamed to report the situation, and believing that reporting was useless [53]. 

Some limitations of our study should be mentioned. A study with a larger sample size would increase the statistical power to identify new associations. Our sample consisted of patients examined during the day (8:30 a.m. to 7:00 p.m.), which could also be another limitation of this study. In this study, only physical, psychological, and sexual violence were assessed. Economic or digital violence are other forms of domestic violence that are equally important and devastating but not examined in the present study, which could be the subject of a future broader study. It should be noted that “verbal abuse” was included here in psychological abuse for simplicity. Moreover, it is worth mentioning that this is an observational study. The collected data are based on self-reported information, left to the patient’s discretion. Indeed, it would not be surprising if some patients who consult a psychiatric emergency room falsely claim that they are victims of violence in order to be hospitalized. However, Howard et al. suggested that this is an underestimation of the data collected [34]. In general, patients who are victims of domestic violence tend to minimize violence due to feeling shame, guilt, hope that the violence is temporary, and other reasons already mentioned [52,53]. There may also be an underestimate of reported violence because patients do not always realize that violence is involved. For some patients with psychiatric pathologies, being abused may seem normal or even justified. This hypothesis is well-illustrated during the collection of complaints from the victims, one of whom explained that she refused to file a complaint because “when I am sick, I am horrible; it is normal for him to hit me”. Taking into account the above-mentioned observations, it is quite probable that the prevalence of domestic violence in the present study is underestimated.

In light of the results of the present study, it seems essential to systematically detect domestic violence in emergency room patients (general and psychiatric) to propose better care and reduce co-morbidities, particularly the risk of suicide. The consultation of the victims through advice and psychological support may encourage them to proceed with the legal procedure. The decision to take legal action may be more difficult for patients with psychiatric disorders due to their emotional state, social isolation, vulnerability, or absence of a guardian. Explaining to these patients the concept of violence and that it is never justified is very important, and forensic medicine units and victims’ aid associations may have a crucial role in their identification and support. 

## 5. Conclusions

The psychiatric population should be identified as a population at risk for domestic violence, which implies the need for systematic screening for abuse. Our study showed a prevalence of 38.2% among patients consulting a French emergency psychiatric department, as well as a higher risk of domestic violence in the female population with previous suicide attempts. A study with a larger sample size could increase the statistical power to probably identify new associations with other psychiatric disorders. The presence of psychiatric pathology appears to be both a consequence and a trigger for domestic violence, and both situations should be detected systematically. Early identification and adequate support of the victims of domestic violence with psychiatric disorders—a quite vulnerable population—should be considered of paramount importance for our society, and targeted efforts must be performed in this direction.

## Figures and Tables

**Table 1:** Socio-demographic characteristics according to reported domestic violence.

	All Patients	DV+	DV−	*p*-Value
Number of patients	152 (100%)	58 (38.2%)	94 (61.8%)	
Mean age	40.2 ± 16.4	41.1 ± 15.1	39.7 ± 17.2	0.592
Mean age—males	42.7 ± 18.0	33.9 ± 9.6	44.6 ± 18.8	**0.029**
Mean age—females	39.1 ± 5.5	42.3 ± 15.5	36.2 ± 15.1	**0.043**
Male	47 (30.9%)	8 (17.0%)	39 (83.0%)	**<0.001**
Female	105 (69.1%)	50 (47.6%)	55 (52.4%)	

Notes. DV+: reported domestic violence. DV−: no domestic violence. Values referred to absolute and relative frequencies (%) or mean ± standard deviation (SD). *p*-value was assessed using *t*-test or Fisher’s exact test. Data in bold: The significant results.

**Table 2:** Details of multiple domestic violence.

	n (% Total of DV+ Cases)
Multiple domestic violence	34 (58.6%)
Psychologic and physical	19 (32.8%)
Psychologic, physical, and sexual	13 (22.4%)
Psychologic and sexual	1 (1.7%)
Physical and sexual	1 (1.7%)

Notes. DV+: reported domestic violence. Values referred to absolute and relative frequencies (%).

**Table 3:** Psychiatric disorders and reported domestic violence according to sex.

	Male			Female		
	DV+	DV−	*p*-Value	DV+	DV−	*p*-Value
Mood disorder	5 (20.8%)	19 (79.2%)	0.701	28 (45.9%)	33 (54.1%)	0.697
Anxiety disorder	0 (0.0%)	5 (100.0%)	0.571	5 (25.0%)	15 (75.0%)	**0.027**
Psychotic disorder	0 (0.0%)	2 (100.0%)	>0.999	0 (0.0%)	2 (100.0%)	0.496
Personality disorder	3 (42.9%)	4 (57.1%)	0.084	9 (52.9%)	8 (47.1%)	0.792
Addiction disorder	2 (18.2%)	9 (81.8%)	>0.999	8 (61.5%)	5 (38.5%)	0.377
Disorders related to trauma and stress	1 (25.0%)	3 (75.0%)	0.539	5 (55.6%)	4 (44.4%)	0.733
Others	0 (0.0%)	1 (100%)	>0.999	1 (33.3%)	2 (66.7%)	>0.999
Suicidal attempt	1 (50.0%)	1 (50.0%)	0.315	8 (80.0%)	2 (20.0%)	**0.045**
No psychiatric diagnosis	0 (0.0%)	5 (100.0%)	0.571	3 (100.0%)	0 (0.0%)	0.105

Notes. DV+: reported domestic violence. DV−: no domestic violence.Values referred to absolute and relative frequencies (%). *p*-value was assessed via Fisher’s exact test. Data in bold: The significant results.

**Table 4:** Multivariate logistic regression of reported domestic violence.

	*p*-Value	OR	95% CI OR
Mood disorder	0.368	0.60	0.20–1.81
Anxiety disorder	0.050	0.26	0.07–1.00
Suicidal attempt	**0.021**	5.29	1.28–21.86
Personality disorder	0.900	1.07	0.38–3.01
Addiction disorder	0.641	0.76	0.25–2.37
Psychotic disorder	0.999	0.00	0.00–0.00
No psychiatric diagnosis	0.553	0.57	0.09–3.64
Disorders related to trauma and stress	0.464	0.54	0.11–2.78
Others	0.489	0.41	0.03–5.20

Notes. Dependent variable: reported domestic violence (yes vs. no). Independent mood disorder, anxiety disorder, suicidal attempt, personality disorder, addiction disorder, psychotic disorder, no psychiatric diagnosis, disorders related to trauma and stress, and others. Values referred to odds ratio (OR) and its 95% confidence interval (CI). Data in bold: The significant results.

## Data Availability

All data generated or analyzed during this study are included in this published article or are available from the corresponding author upon reasonable request.
